# FokI Polymorphism of the *VDR* Gene Is Associated with Vitamin D Insufficiency in Elite Male Power Athletes of Kazakhstan

**DOI:** 10.3390/nu17203195

**Published:** 2025-10-11

**Authors:** Aidana Gabdulkayum, Saya Amangeldikyzy, Adil Yerezhepov, Sayipzhamal Khassanova, Kenes R. Akilzhanov, Ulan Kozhamkulov, Saule Rakhimova, Ulykbek Kairov, Ainur Akilzhanova, Dauren Yerezhepov

**Affiliations:** 1 Laboratory of Genomic and Personalized Medicine, Center for Life Sciences, National Laboratory Astana, Nazarbayev University, Astana 010000, Kazakhstan; 2 The National Center for Children’s Rehabilitation, Astana 010000, Kazakhstan; 3 Department of Biotechnology, Al-Farabi Kazakh National University, Almaty 050040, Kazakhstan; 4 The National Center for Sports Medicine and Rehabilitation, Almaty 020000, Kazakhstan; 5 Department of Medicine, Semey Medical University, Pavlodar Branch, Pavlodar 140000, Kazakhstan; 6 Laboratory of Bioinformatics and Systems Biology, Center for Life Sciences, National Laboratory Astana, Nazarbayev University, Astana 010000, Kazakhstan

**Keywords:** vitamin D deficiency, vitamin D receptor, power athletes, VDR FokI polymorphism

## Abstract

**Background/Objectives**: We aimed to investigate the association between *VDR* gene variants and vitamin D levels in elite male power athletes of Kazakhstan. **Methods**: We recruited 92 elite male power athletes of Kazakhstan. Concentrations of serum 25(OH)D were measured with the Access 25(OH) Vitamin D Total Assay on the Unicel Dxl 800 Access Immunoassay System. Gene polymorphisms were determined by a real-time polymerase chain reaction (RT-PCR) allelic discrimination assay using TaqMan™ probes. **Results**: Vitamin D insufficiency was registered in 63% of athletes. Age (χ^2^ = 6.83, *p* < 0.01), BMI (χ^2^ = 6.83, *p* < 0.01), and sport experience (χ^2^ = 4.44, *p* < 0.04) showed a statistically significant association with vitamin D insufficiency and deficiency (age, χ^2^ = 7.93, *p* < 0.01; BMI, χ^2^ = 5.11, *p* < 0.03; sport experience, χ^2^ = 6.19, *p* = 0.01). The A/A genotype of the *VDR* FokI polymorphism (rs2228570) showed a strong correlation with vitamin D insufficiency (G/G-G/A vs. A/A, OR = 9.25, 95% CI = 2.01–42.51, *p* < 0.01) but not deficiency. **Conclusions**: Our study reveals a significant prevalence of vitamin D insufficiency and deficiency among elite male power athletes of Kazakhstan. Age, BMI, and sport experience are essential factors in developing personalized strategies to address vitamin D insufficiency. The A/A genotype of the *VDR* FokI polymorphism can be used as a potential biomarker for vitamin D inadequacy in elite male power athletes of Kazakhstan.

## 1. Introduction

The scientific history of vitamin D spans over a century. A bone disease caused by vitamin D deficiency, rickets, was the reason for the discovery of vitamin D. Cod liver oil was the source where vitamin D was first discovered as a newly identified antirickets factor. Since it was discovered after fat-soluble factor A (vitamin A), water-soluble factors (vitamin B), and anti-scurvy factor (vitamin C), it was named vitamin D. Since then, enormous research efforts have been dedicated to vitamin D. Now its deficiency is recognized as a health condition [1].

Vitamin D plays a crucial role in calcium and phosphorus metabolism, as well as in bone formation and overall health [2]. However, the main mediator of vitamin D action, vitamin D receptor (VDR), has been found in many tissue types and it is involved in many non-skeletal-function-related processes like immune response and inflammation, cardiovascular events, metabolic syndrome and diabetes, cancers, and mood disorders [3,4] and regulates the expression of almost a thousand genes [5]. Many research studies have reported that optimal serum 25-hydroxyvitamin D [25(OH)D] levels are positively correlated with sports performance, strength, power, endurance, aerobic abilities, fracture risk, and faster recovery [6,7,8,9].

The prevalence of vitamin D deficiency in the general population is high. However, its indicators vary between studies since the serum concentration of 25-hydroxy-vitamin D [25(OH)D] depends on season and latitude, sun exposure and the use of sun protectors, skin tone and dietary habits [6]. Vitamin D deficiency is similarly high in elite athletes. Due to variability in vitamin D levels and demands for different sports types, there is no special vitamin D level threshold for athletes; thus, deficiency is defined as <19.99 ng/mL, insufficiency at 20–29.99 ng/mL, and sufficiency ≥30 ng/mL according to the Consensus Statement on vitamin D status for the general population [3].

Most vitamin D is synthetized in skin during exposure to ultraviolet B (UVB) rays of sunlight [10]. Newly synthetized vitamin D enters the bloodstream, binds with the vitamin D binding protein (VDPB), and travels to the liver. In the liver, it is hydroxylated to 25-hydroxyvitamin D. In the kidney, 25(OH)D undergoes further hydroxylation to form the biologically active metabolite 1,25-dihydroxyvitamin D [1,25(OH)_2_D]. To perform its action, it must enter the cell, which is mediated by VDR [11]. VDR is a transcriptional modulator that is found on the surface of cells of almost every tissue and is encoded by the *VDR* gene. When Miyamoto et al. described the structure of the *VDR* gene [12], many researchers drew their attention to *VDR* gene variations and their association with vitamin D metabolism and many health conditions [13,14,15,16,17]. Several research studies related to sports medicine reported a correlation between *VDR* gene polymorphisms and performance and injuries in athletes [18,19,20,21].

Power sports athletes are individuals who participate in sports that require a high level of explosive strength, speed, and power. These athletes rely on their ability to generate force quickly and efficiently, often with bursts of energy, and train with high resistive muscle loads [22]. Such demands in terms of strength, speed, and power require rapid calcium transportation to increase muscle contractions. Vitamin D is a potent modulator of skeletal muscle physiology by activating the expression of genes involved in calcium and phosphate transport by muscles through cell membranes, phospholipid metabolism, muscle growth, and differentiation. Athletes with optimal vitamin D levels exhibit higher performance levels and faster rehabilitation and recovery from injuries, as vitamin D deficiency is strongly associated with myopathy [23].

The present research aims to determine the association between the most studied *VDR* variants (TaqI rs731236, ApaI rs7975232, BsmI rs1544410, and FokI rs2228570) and the serum concentration of 25-hydroxy-vitamin D [25(OH)D] in elite male power athletes of Kazakhstan.

## 2. Materials and Methods

### 2.1. Study Subjects

We recruited active athletes who were included in the main and reserve National Olympic Team of Kazakhstan in various power sports. After explaining the study’s aim and protocol, all subjects were given an informed consent form for review. We proceeded with the interviews and blood sampling only after obtaining informed consent.

Athletes who had supplements containing any vitamin D (including vitamin D-containing multivitamins) six months before recruitment were excluded from the study to avoid intervention in serum vitamin D levels. Since seasonal serum vitamin D levels may vary due to territorial reasons, recruitment was performed in the second part of the summer between late July and August 2024, as Kazakhstan is located between 40′ and 60′ latitude of the northern hemisphere, with long winters and a low UV index. At this time of the season, the UV index is high in most of Kazakhstan’s territory, and the natural vitamin D level reaches its maximum.

The recruitment took place in the training camps AIBA (Almaty region, South-East Kazakhstan) and Jaksy-2 (Borovoe, Northern Kazakhstan). Eligible participants filled out structured sociodemographic and clinical questionnaires during an in-person interview. The interview and collection of anthropometric measurements, including height, weight, and body mass index (BMI), were performed by sports medicine specialists of the Center for Sports Medicine and Rehabilitation “ProSport” (Astana, Kazakhstan) and National Center for Sports Medicine and Rehabilitation (Almaty, Kazakhstan). Dietary data were taken from the dietary protocol of the National Olympic Committee of Kazakhstan for the year. All athletes consumed oily fish once a week, ensuring that the dietary intake of vitamin D-rich foods was equal for all participants.

This study was conducted in accordance with the Declaration of Helsinki and approved by the Local Ethics Committee of the Private Institution “National Laboratory Astana” (Protocol N05-2022, 21 October 2022). All subjects signed a written informed consent form. 

### 2.2. Blood Sampling

Blood sampling was performed between 7:00 and 9:00 a.m. after overnight fasting. Blood was drawn into a blood collection tube containing K2EDTA for DNA isolation (BD, Franklin Lakes, NJ, USA). For biochemical analysis, blood was drawn into a blood collection tube coated with micronized silica particles to accelerate clotting and containing a polymer gel for serum separation (BD, NJ, USA). Blood collection tubes were centrifuged for 10 min at 2000 rpm. The serum was aliquoted for 500 µL per tube and stored at −80 °C until used for biochemical analysis.

### 2.3. Biochemical Analysis

All serum samples passed one freeze–thaw cycle before measuring the serum 25(OH)D levels. Concentrations of serum 25(OH)D were measured with the Access 25(OH) Vitamin D Total Assay on the Unicel Dxl 800 Access Immunoassay System (Beckman Coulter, Brea, CA, USA). The Access 25(OH) Vitamin D Total assay (Beckman Coulter, Brea, CA, USA) is a solid-phase Enzyme Linked Immunosorbent Assay performed on specific monoclonal antibody-linked microtiterplates. The amount of substrate turnover is determined by measuring the absorbance, which is inversely proportional to the total 25(OH)D concentration. Concentrations of serum 25(OH)D were measured in laboratories utilizing the Vitamin D Standardization Program (VDSP), which was established in 2010 by the National Institutes of Health Office of Dietary Supplements, Centers for Disease Control and Prevention (CDC), National Institute of Standards and Technology (NIST), and Ghent University [24]. The traceability and harmonization of the Access 25(OH)D Vitamin D Total assay is performed according to SRM 2972 and has a total imprecision of ≤10.0% CV at concentrations > 15.0 ng/mL (37.5 nmol/L) and standard deviation (SD) ≤ 1.5 ng/mL (3.8 nmol/L) at concentrations ≤ 15.0 ng/mL. The Unicel Dxl 800 Access Immunoassay System (Beckman Coulter, Brea, CA, USA) passes calibration using Access 25(OH) Vitamin D Total Calibrators (A98856, Beckman Coulter, Brea, CA, USA) every 24 h, and service procedures and maintenance are performed according to the schedule or as needed.

### 2.4. DNA Isolation

DNA was extracted from 300 μL of whole blood using the Illustra Blood Genomic Prep Spin Kit (Cytivia, Marlborough, MA, USA), according to the manufacturer’s instructions, and stored at −20 °C. The quality and quantity of extracted gDNA were assessed by the spectrophotometric method using the NanoDrop 2000 UV spectrophotometer (ThermoFisher Scientific, Waltham, MA, USA) and fluorometric assay using the Qubit BR Assay Kit (ThermoFisher Scientific, Waltham, MA, USA) on a Qubit v4.0 fluorometer. To test the integrity of the isolated DNA, 1% agarose gel electrophoresis was used. DNA was stored at −20 °C in the Laboratory Biobank.

### 2.5. Genotyping

In this study, we have chosen the four most extensively researched gene polymorphisms: ApaI (rs7975232), BsmI (rs1544410), FokI (rs2228570), and TaqI (rs731236). Selected polymorphisms of the *VDR* gene are presented in Table 1.

The gene polymorphisms were determined using a real-time polymerase chain reaction (PCR) allelic discrimination assay using TaqMan™ probes (Applied Biosystems, Foster City, CA, USA) on the 7900 HT Fast Real-Time PCR System following the manufacturer’s protocol. The 10 uL reaction mixture consisted of the 5 uL of 2× TaqMan™ Genotyping master mix (Applied Biosystems, Foster City, CA, USA), 0.25 uL of 40× (catalogue number: 4351379, Applied Biosystems, Foster City, CA, USA) or 0.5 uL of 20× (catalogue number: 4331349, Applied Biosystems, Foster City, CA, USA) TaqMan™ Probe, and ten ng of genomic DNA.

The genotype distribution was analyzed using SDS v2.4 software (Applied Biosystems, Foster City, CA, USA). Each genotyping was repeated three times to avoid mistakes during software distribution. Minor allele frequencies (MAFs) of less than 0.05 were considered rare, and MAF under 0.01 was considered very rare.

### 2.6. Statistical Analysis

Quantitative variables were expressed as a result (±standard deviation) with a normal distribution. We applied the Hardy–Weinberg equilibrium test for all subjects. We analyzed 2 × 2 associations between vitamin D status and epidemiological indicators using the Pearson χ^2^ test. Binary logistic regression analysis was used to evaluate the association between vitamin D status and epidemiological data. The association between vitamin D status and studied polymorphisms was evaluated using multimodal (genotypic, dominant, recessive, and overdominant) logistic regression adjusted for age, BMI, and sports experience and assessed with the ORs and their corresponding 95% CIs. We defined the models as follows: genotypic (AA vs. Aa vs. aa), dominant (AA + Aa vs. aa), recessive (AA vs. Aa + aa), and overdominant (AA + aa vs. Aa). The major and the minor alleles are denoted as A and a, respectively. All tests were two-sided, with a significance level of *p* < 0.05, and were analyzed using SPSS 25 (IBM, Armonk, NY, USA) software.

## 3. Results

### 3.1. Study Group

**Figure 1:**
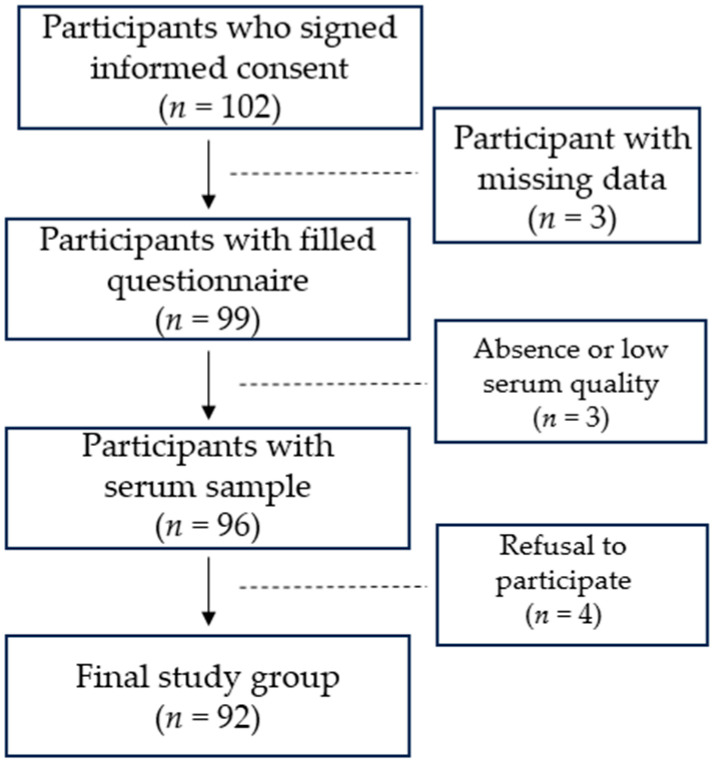
Illustration of the study design.

A hundred and two active elite power male athletes who were included in the main and reserve National Olympic Team of Kazakhstan in various sports types were recruited between July and August 2024. Three participants were excluded due to missing data, including information on sporting experience or vitamin D supplementation, as reported in the questionnaire. Three participants were excluded due to low serum quality. Four athletes refused to participate in the study in the later stages of the research. The final study group for association analysis involving serum 25(OH)D levels consisted of 92 samples. The study design is presented in Figure 1.

All athletes were at least candidates for the Master of Sports or Master of Sports of International Class. Many of them participated in the World, European, and Asian championships and won various medals. Recruited athletes represented the following power sports types: boxing (30), judo (21), freestyle wrestling (14), taekwondo (12), Greco-Roman wrestling (9), and weightlifting (6).

During the active training periods (11 months per year), elite athletes are located in the training camps. According to the dietary and training recommendations of the Ministry of Tourism and Sports of the Republic of Kazakhstan, the diet of all elite athletes is unified and includes the consumption of oily fish at least once a week to maintain a balanced diet.

Due to Kazakhstan’s climatic conditions, outdoor training for indicated sport types lasts one to two hours during the warm periods from May to October. The rest of the time, training takes place indoors as the temperature drops to −15 °C during the fall and up to −40 °C during the winter. The training program for power sports during summer camps includes one hour of outdoor running and workouts, with up to three sessions a day.

The mean age was 24.5 ± 4.1 years, the median height was 177.2 ± 9.3 cm, the mean weight was 82.1 ± 22.8 kg, and the average BMI was 25.9 ± 5.5 kg/m^2^. The mean sporting experience was 13.1 ± 4.2 years, with participants who trained for over or under thirteen years being equally distributed. Demographic and epidemiological data of study participants are shown in Table 2. Detailed data on vitamin D levels, age, BMI, and years of training for individual sports types is presented in the Supplementary Material.

### 3.2. Serum Concentration of 25-Hydroxy-Vitamin D [25(OH)D]

**Figure 2:**
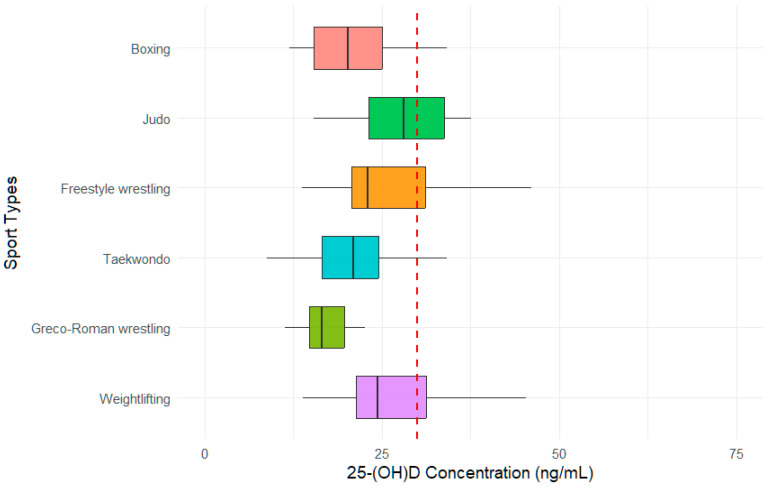
The vitamin D status of study participants by sport types (n = 92). The threshold is represented by a red dotted line and set at 30 ng/mL.

Overall, 92 samples were sent to identify serum 25(OH)D levels. The mean serum 25(OH)D level of the study group was 24.4 ± 8.7 ng/mL. The distribution of vitamin D status by sports type is presented in Figure 2. Athletes representing boxing, taekwondo and Greco-Roman wrestling had low median serum 25(OH)D levels, while athletes of judo showed higher median levels. The vitamin D status of the study participants is shown in Table 3.

Almost two-fifths (38%) of study participants had vitamin D deficiency, a quarter had vitamin D insufficiency, and 34 athletes (37%) were vitamin D sufficient, meaning 63.0% of athletes had vitamin D inadequacy.

### 3.3. Association of Vitamin D Status with Epidemiological Data of Study Participants

We assessed the association of vitamin D status with epidemiological data such as age, BMI, and sports experience. Since the mean age was 24.5 years and the mean sports experience was 13.1 years, we set separation thresholds of 25 years and 13 years for age and sports experience, respectively. The separation threshold for BMI was set at 25 kg/m^2^. An association analysis demonstrated that age (χ^2^ = 9.26, *p* < 0.01), BMI (χ^2^ = 7.04, *p* < 0.03), and sports experience (χ^2^ = 6.84, *p* = 0.03) showed a statistically significant correlation with vitamin D status in our study cohort of athletes. The results indicate that age, BMI, and sports experience can affect the vitamin D status in our cohort. The association of vitamin D status with epidemiological data of study participants is presented in Table 4.

Furthermore, we examined the association of vitamin D deficiency, insufficiency, and sufficiency with epidemiological data in the following manner: deficiency/insufficiency vs. sufficiency and deficiency vs. insufficiency/sufficiency. Binary logistic regression analysis showed that age (<25 vs. ≥25, OR = 3.18, 95% CI = 1.32–7.66, *p* < 0.01) and BMI (<25 vs. ≥25, OR = 3.18, 95% CI = 1.32–7.66, *p* < 0.01) were significantly associated with vitamin D insufficiency and deficiency (age, <25 vs. ≥25, OR = 3.75, 95% CI = 1.46–9.65, *p* < 0.01; BMI, <25 vs. ≥25, OR = 2.78, 95% CI = 1.13–6.82, *p* < 0.03). Sport experience showed a statistically significant association with vitamin D sufficiency in both models (<30 vs. ≥30, OR = 0.38, 95% CI = 0.15–0.94, *p* < 0.04; <20 vs. ≥20, OR = 0.33, 95% CI = 0.13–0.80, *p* < 0.02). According to the data, male athletes under 25 years old with a BMI lower than 25 kg/m^2^ and sport experience less than 13 years are likely to be vitamin D-deficient or at least insufficient. The distribution of vitamin D deficiency, insufficiency, and sufficiency among study participants, categorized by epidemiological characteristics, is presented in Table 5.

### 3.4. Genotyping Samples for Polymorphisms of the VDR Gene

We successfully genotyped all 92 samples for all four polymorphisms. Each polymorphism was performed in three repeats. The minor G/G allele of the *VDR* TaqI polymorphism was found only in three athletes (3.3%), and the minor C/C allele of the *VDR* BsmI polymorphism was found only in four athletes (4.4%). The minor alleles of ApaI and FokI polymorphisms accounted for more than 10%. The observed genotype frequencies did not deviate from the expected frequency values according to the Hardy–Weinberg equilibrium test for each comparison (for total observation and groups separately, *p* > 0.05, Supplementary Material). Genotyping results on polymorphisms of selected genes are presented in Table 6.

### 3.5. Genetic Association of Polymorphisms of the VDR Gene with Vitamin D Status

We used the deficiency/insufficiency vs. sufficiency and deficiency vs. insufficiency/sufficiency models to investigate the association between the *VDR* gene polymorphisms and the vitamin D status of the study group. TaqI, BsmI, and ApaI polymorphisms of the *VDR* gene did not show a statistically significant association with vitamin D status.

TaqI, BsmI, and ApaI polymorphisms did not show a statistically significant association with vitamin D status. The A/A genotype of *VDR* FokI showed a statistically significant association with vitamin D insufficiency in the codominant model (G/G vs. G/A vs. A/A, OR = 8.90, 95% CI = 1.80–43.97, *p* < 0.01). In the same model, the heterozygous G/A genotype of the same polymorphism showed a protective effect (G/G vs. A/G vs. A/A, OR = 0.92, 95% CI = 0.31–2.67, *p* < 0.01). In the recessive model, the A/A genotype of the VDR FokI polymorphism showed a strong correlation with vitamin D insufficiency (G/G-G/A vs. A/A, OR = 9.25, 95% CI = 2.01–42.51, *p* < 0.01). However, the FokI polymorphism of the *VDR* gene did not show a statistically significant association with vitamin D deficiency. The genetic association of polymorphisms of the *VDR* gene with vitamin D status is presented in Table 7.

## 4. Discussion

Vitamin D is a biologically inert molecule, and its activation requires two successive hydroxylation reactions to synthesize its biologically active form, 1,25-dihydroxyvitamin D, which is essential for many vital functions in overall health [17,25]. Numerous research studies have reported its deficiency in the general population [2,3,4] and a wide range of health conditions and diseases [17,26,27,28]. Serum 25(OH)D levels depend on age, latitude, season, the amount of exposure to the sun, skin color, diet, a sedentary lifestyle, and genetics [29]. Populations of Kazakhstan historically have many geographical conditions to be vitamin D-deficient. These conditions include being between 40′ and 60′ latitude of the northern hemisphere, a continental climate with long winters and short summers in most of its territory, no direct access to the sea, and vitamin D-rich food. Yerezhepov et al. reported a high prevalence of vitamin D deficiency in healthy controls, who were recruited during the summer period [17]. Another cross-sectional study by Gromova et al. also found a high prevalence of vitamin D deficiency in a healthy population of Kazakhstan [30].

Vitamin D status is a hot topic in sports science since it can affect musculoskeletal function, injury risk, and performance [2,25,31,32]. In our study, 38% of athletes were vitamin D-deficient, 25% were in the range of insufficiency, and 37% had a sufficient level of 25(OH)D. Inadequacy constituted 63% in the summer period, when serum vitamin D levels are highest. Many research studies have reported a high prevalence of vitamin D insufficiency and deficiency among athletes [6,25,33,34,35,36,37]. A systematic review and meta-analysis performed by Harju et al. reported a mean serum 25(OH)D concentration of 66.4 nmol/L (26.6 ng/mL), which was pooled from 3725 athletes in 27 studies. They reported that the prevalence of vitamin D insufficiency among eligible adult studies was 30% and it was 39% among adolescent athletes [6]. Hacker et al. found that more than half of 474 German athletes had insufficient serum concentration levels of 25-hydroxy-vitamin D [25(OH)D] and 16% had deficient serum concentration levels of 25-hydroxy-vitamin D [25(OH)D] [33]. Bezuglov et al. found that out of 131 young Russian male footballers, more than 40% had inadequate vitamin D levels [34]. Grieshober et al. demonstrated that over 70% of National Basketball Association players were vitamin D-insufficient [35]. A systematic review and meta-analysis by Farrokhyar et al. reported a 56% prevalence of vitamin D inadequacy in 2313 athletes, with higher levels in the UK and the Middle East. They also found that the risk is significantly increased seasonally and at latitudes greater than 40° N [36]. Hamilton et al. reported that 84% of professional football players of different origins had vitamin D deficiency [37]. Our findings confirm the results of Ip et al. They reported that vitamin D inadequacy is more prevalent in power sports (65.4%) than endurance sports (32.9%) [25]. Mehran et al. reported interesting results. They demonstrated that in their study cohort, vitamin D-deficient athletes were absent, and only more than 10% had vitamin D inadequacy. However, 96% of their studied hockey players were Caucasians [38]. This is not a surprising, given that vitamin D deficiency is prevalent in Asian populations [39,40]. Sutherland et al. demonstrated that the prevalence of vitamin D deficiency was highest among participants of Asian ancestry (57.2% in winter/spring and 50.8% in summer/autumn) [39]. Shraim et al. reported that 9% of Asians were profoundly vitamin D-deficient, while 47% were severely deficient [40]. In addition, we found a correlation between vitamin D status and age, BMI, and sports experience. In our study cohort, males under 25 years with a BMI lower than 25 kg/m2 and sporting experience of less than 13 years were more likely to have vitamin D inadequacy. The reason may lie in the metabolic properties of a young athlete’s body. A meta-analysis of elite athletes reported that more than one-third of adolescent elite athletes in their study were vitamin D-insufficient [6]. The association between vitamin D status and BMI is inconsistent. Many research works have reported that a higher BMI is associated with vitamin D insufficiency. Due to its lipophilic nature, newly synthesized vitamin D is accumulated in adipose tissue (the main storage site of vitamin D in the body) and its availability decreases. In addition, its release from adipose tissue is slow and requires physical activity or exercise [25]. During the training process, athletes may burn fat, and this may lead to an increase in serum vitamin D levels. Young and adolescent athletes may have lower fat content, which could be an explanation for the higher prevalence of vitamin D insufficiency among this population.

Skin exposure to UVB radiation is the only natural way to synthetize vitamin D in the human body endogenously [2]. During skin exposure to sunlight, a precursor of vitamin D is synthetized by photochemical reaction [1]. Since this reaction produces most of the vitamin D, time being under sunlight is crucial for serum vitamin D levels. Many studies have been reported of the effect of the training environment on the serum vitamin D levels [41,42]. A systematic review and meta-analysis of Barsan et al. reported a difference in the level of vitamin D between indoor and outdoor athletes [41]. Valtueña et al. reported that 82% of 408 elite athletes had vitamin D inadequacy, and outdoor-trained athletes had higher vitamin D levels than indoor-trained athletes [42]. However, to avoid alterations and biases in vitamin D levels, we recruited elite male power athletes with similar training regimens.

Maintaining adequate levels of vitamin D is essential, especially for athletes. Therefore, sports nutritionists and physicians must regularly evaluate serum vitamin D levels in athletes. Insufficiency for athletes is considered to be less than 30 ng/mL, as for the general population. However, Yoon et al. suggested keeping serum vitamin D levels above 32 ng/mL and preferably not lower than 40 ng/mL [43]. Nutritionists recommend approximately 30 min of daily sun exposure and a vitamin D-rich diet [23,44]. However, there are many natural factors, such as season, latitude, genetics, and anthropogenic factors like urbanization, air pollution, diet, sunburn avoidance, and the use of sunscreens, as well as a sedentary lifestyle, that have an adverse effect on the level of vitamin D [45,46]. Due to various factors, the recommendations of nutritionists may not be sufficient to meet the demands of an athlete. In such cases, vitamin D supplements may be helpful [43]. Vitamin D supplementation has shown beneficial effects in numerous studies for athletes [8,47,48,49]. Shuler et al. reported a minimal concentration of 40 ng/mL for reducing stress fractures and no apparent benefit from 25(OH)D levels above 50 ng/mL [8]. Bolland et al. stated that there is little justification to use vitamin D supplements to maintain or improve musculoskeletal health [47]. Jung et al. reported that vitamin D supplementation reduces the symptoms of upper respiratory tract infection during winter training in taekwondo athletes [48]. Żebrowska et al. reported that three weeks of vitamin D supplementation had a positive effect on serum 25(OH)D levels in endurance-trained runners [49]. For the general population, the recommended dose of vitamin D supplements is 400–600 IU to maintain serum vitamin D levels [2]. There are no recommended screening guidelines for vitamin D deficiency designated for athletes. In a study by Michalczyk et al., professional soccer players with vitamin D insufficiency significantly increased their blood levels of 25(OH)D after 6000 IU of daily vitamin D supplementation for 6 weeks [50]. In general, a daily vitamin D supplementation of 2000–6000 IU is recommended for athletes [8,44,47,48,50]. However, athletes may require higher doses of vitamin D supplements in some exceptional cases, like severe deficiency [43,51]. It is clearly seen that athletes in our study cohort require vitamin D supplementation for injury prevention and performance improvement, as inadequacy was registered in 63% of participants, with serum samples collected during summer.

The metabolic pathway of vitamin D comprises multiple steps and involves many products of many genes [52,53]. Sepulveda-Villegas et al. reported that 35 genes are associated with vitamin D deficiency, and 12 of them were directly involved in vitamin D metabolism [52]. Jolliffe et al. reported that more than 50 SNPs in genes related to the vitamin D pathway are correlated with concentrations of vitamin D in the serum [53]. However, the primary mediator, VDR, takes center stage. The human *VDR* gene is located on chromosome 12 and contains 11 exons [12]. Currently, researchers have reported almost 1000 allelic variants in the VDR locus [54]. Taql (rs731236), Apal (rs7975232), BsmI (rs1544410), and Fokl (rs10735810) polymorphisms have been studied most extensively since they were found first. Taql, Apal, and BsmI polymorphisms cause silent mutations at the 3′-untranslated region (UTR) of the *VDR* gene. Some studies suggest they are responsible for mRNA expression and stability. The first three exons of the *VDR* gene are located at 5′-UTR, and the Fokl (rs10735810) polymorphism, which is located on exon 2, shifts the start codon. As a result, an altered version of mRNA is synthetized and a shortened protein is produced [55,56]. In our study, we found a statistically significant association of *VDR* FokI polymorphism with inadequate levels of vitamin D. The A/A genotype of *VDR* FokI showed a statistically significant association with vitamin D insufficiency (G/G vs. G/A vs. A/A, OR = 8.90, 95% CI = 1.80–43.97, *p* < 0.01) and the heterozygous G/A genotype showed a protective effect (G/G vs. A/G vs. A/A, OR = 0.92, 95% CI = 0.31–2.67, *p* < 0.01). In the recessive model, the A/A genotype of the *VDR* FokI polymorphism showed a strong correlation with vitamin D insufficiency (G/G-G/A vs. A/A, OR = 9.25, 95% CI = 2.01–42.51, *p* < 0.01). Many studies have reported an association between *VDR* gene polymorphisms and serum vitamin D levels [13,57,58,59,60,61,62,63]. AbdElneam et al. found an association between the TaqI genotype and increased serum vitamin D levels in patients with mild to moderate psoriasis vulgaris [57]. Gaffney-Stomberg et al. reported that the BsmI (rs1544410) polymorphism of the *VDR* gene was associated with a higher circulating 25(OH)D concentration [58]. Hibler et al. found an association between serum vitamin D levels and SNPs of the *VDR* and *RXRA* genes [13]. In a meta-analysis by Krasniqi et al., the authors report several studies that demonstrate an association between *VDR* gene polymorphisms and serum vitamin D levels [59]. Orton et al. investigated the genetic contribution to 25(OH)D concentrations in twins with multiple sclerosis. They reported that the mean concentration in Canadian subjects with the FokI genotype (GG) coding for the shorter length *VDR* was 64.6 ± 5.6 nmol/L compared with 83.2 ± 3.9 nmol/L in subjects with the AA and AG genotypes [60]. Lazaro et al. reported similar results in CrossFit practitioners. They found that the homozygous G/G genotype of the *VDR* FokI polymorphism had higher levels of circulating vitamin D compared to individuals with the AA genotype [61]. Tanabe et al. reported a positive association between the *VDR* FokI (rs2228570) polymorphism and serum 25(OH)D levels in a Japanese healthy population [62]. Sadat-Ali et al. found that vitamin D deficiency in Saudi Arabians was associated with SNP rs2228570 of the *VDR* gene [63].

There is no clear evidence of a direct influence of the *VDR* gene on the concentrations of 25(OH)D in serum since it binds to an active form of vitamin D, 1,25-dihydroxyvitamin D. Many assays are designed to detect the form of vitamin D, 25(OH)D that undergoes one-time hydroxylation in the liver by hepatic 25-hydroxylase, which is encoded by cytochrome P450 mixed-function oxidases (CYPs; CYP27A1 encodes the mitochondrial 25-hydroxylase, and CYP2R1 is identified in the microsomal fraction of the liver). CYP27A1 and CYP2R1 are interconnected. In a CYP27A1-null mouse, the expression of CYP2R1 is increased and leads to elevated blood levels of 25(OH)D in these mice. The blood levels of 25(OH)D in mice with the deleted CYP2R1 gene decreased more than 50% [2]. Therefore, genetic variation in the VDR gene may lead to the enhanced absorption of 1,25(OH)_2_D and suppress the expression of CYPs in the liver, resulting in vitamin D circulating in the bloodstream as a vitamin D–VDBP complex.

*Limitations*

There are some limitations in our study to bear in mind. First is the small cohort size. However, our cohort represents the majority of Kazakhstani elite athletes involved in power sports. Second, vitamin D results represent a snapshot, an indicator at a particular time, and do not represent year-round vitamin D status. However, there is a high level of vitamin D inadequacy among athletes, even though blood samples were collected during July and August. The third limitation concerns outdoor training and the dietary intake of vitamin D. These factors can influence vitamin D status. However, we included sport types with similar training regimes and accessed the dietary data from the dietary protocol of the Ministry of Tourism and Sports of the Republic of Kazakhstan. Fourth, we were unable to collect all necessary anthropometric data such as waist and hip circumference to measure WHR, which complements the BMI data. Fifth, the inclusion of a comparison with a non-athlete group would have strengthened this study and revealed distinctions between these populations. In future investigations, all limitations will be acknowledged and addressed.

*Future Research Directions*

Multiple factors, including genetic, environmental, and lifestyle components, influence vitamin D status. While our study focused on the *VDR* FokI polymorphism, future investigations should expand on this to explore the role of additional genetic variants that regulate vitamin D metabolism and activity. Polymorphisms in genes like *PTH* and *CYP* family members should be examined further in Kazakhstani athletes, as they potentially contribute to the variability in vitamin D status according to the literature.

## 5. Conclusions

Our study reveals a significant prevalence of vitamin D insufficiency and deficiency among elite male power athletes of Kazakhstan. Age, BMI, and sport experience are important factors in developing personalized strategies to address vitamin D insufficiency. The A/A genotype of the *VDR* FokI polymorphism can be used as a potential biomarker for vitamin D inadequacy in elite male power athletes of Kazakhstan.

## Figures and Tables

**Table 1:** Selected polymorphisms of the *VDR* gene.

Gene	Name	Reference Number	Context Sequence
*VDR*	TaqI	rs731236	TGGACAGGCGGTCCTGGATGGCCTC[A/G] ATCAGCGCGGCGTCCTGCACCCCAG
	BsmI	rs1544410	GAGCAGAGCCTGAGTATTGGGAATG[T/C] GCAGGCCTGTCTGTGGCCCCAGGAA
	ApaI	rs7975232	AAGGCACAGGAGCTCTCAGCTGGGC[A/C] CCTCACTGCTCAATCCCACCACCCC
	FokI	rs2228570	GGAAGTGCTGGCCGCCATTGCCTCC[A/G] TCCCTGTAAGAACAGCAAGCAGGCC

VDR: vitamin D receptor.

**Table 2:** Demographic and epidemiological data of study participants (*n* = 92).

Details	All (*n* = 92)
Age, mean ± SD, years	24.5 ± 4.1
Age, years, *n* (%)	
<25	54 (58.7)
≥25	38 (41.3)
Weight, mean ± SD, kg	82.1 ± 22.8
Height, mean ± SD, cm	177.2 ± 9.3
BMI, mean ± SD, kg/m^2^	25.9 ± 5.5
BMI, cat., *n* (%)	
18.5 ≥ <25	48 (52.2)
≥25	44 (47.8)
Sports Experience, mean ± SD, years	13.1 ± 4.2
Sports Experience, years, *n* (%)	
≤13	46 (50.0)
>13	46 (50.0)
Sport types	*n* (%)
Boxing	32 (34.8)
Judo	23 (25.0)
Freestyle Wrestling	14 (15.2)
Taekwondo	10 (10.9)
Greco-Roman Wrestling	9 (9.8)
Weightlifting	4 (4.3)

BMI: body mass index; SD: standard deviation.

**Table 3:** The vitamin D status of study participants (*n* = 92).

Details	All (*n* = 92)
Serum 25(OH)D, mean ± SD, ng/mL	24.4 ± 8.7
Serum 25(OH)D, deficiency vs. insufficiency vs. sufficiency, ng/mL	*n* (%)
<20	35 (38.0)
20 ≥ <30	23 (25.0)
≥30	34 (37.0)
Serum 25(OH)D, deficiency/insufficiency vs. sufficiency, ng/mL	*n* (%)
<30	58 (63.0)
≥30	34 (37.0)
Serum 25(OH)D, deficiency vs. insufficiency/sufficiency, ng/mL	*n* (%)
<20	35 (38.0)
≥20	57 (62.0)

SD: standard deviation.

**Table 4:** Association of vitamin D status with epidemiological data of study participants (*n* = 92).

Variables		Serum 25(OH)D Levels, ng/mL			χ^2^	*p*-value
	Indicators	<20 (*n* = 35)	20 ≥ <30 (*n* = 23)	≥30 (*n* = 34)		
		*n* (%)	*n* (%)	*n* (%)		
Age, years	<25	27 (77.1)	13 (56.5)	14 (41.2)	9.26	<0.01
	≥25	8 (22.9)	10 (43.5)	20 (58.8)		
BMI, kg/m^2^	18.5 ≥ <25	25 (71.4)	15 (65.2)	14 (41.2)	7.04	<0.03
	≥25	10 (28.6)	8 (34.8)	20 (58.8)		
Experience, years	≤13	11 (31.4)	12 (52.2)	19 (63.3)	6.84	0.03
	>13	24 (68.6)	11 (47.8)	11 (36.7)		

**Table 5:** Epidemiological factors associated with vitamin D deficiency, insufficiency, and sufficiency (*n* = 92).

Variables	Indicators	Serum 25(OH)D Levels, ng/mL		OR (95% CI)	*p*-value	Serum 25(OH)D Levels, ng/mL		OR (95% CI)	*p*-value
		<30 (*n* = 58)	≥30 (*n* = 34)			<20 (*n* = 35)	≥20 (*n* = 57)		
		*n* (%)	*n* (%)			*n* (%)	*n* (%)		
Age, years	<25	40 (69.0)	14 (41.2)	3.18 (1.32–7.66)	<0.01	27 (77.1)	27 (47.4)	3.75 (1.46–9.65)	<0.01
	≥25	18 (31.0)	20 (58.8)			8 (22.9)	30 (52.6)		
BMI, kg/m^2^	<25	40 (69.0)	14 (41.2)	3.18 (1.32–7.66)	<0.01	25 (71.4)	27 (47.4)	2.78 (1.13–6.82)	<0.03
	≥25	18 (31.0)	20 (58.8)			10 (28.6)	30 (52.6)		
Experience, years	≤13	23 (39.7)	19 (63.3)	0.38 (0.15–0.94)	<0.04	11 (31.4)	31 (54.4)	0.33 (0.13–0.80)	<0.02
	>13	35 (60.3)	11 (36.7)			24 (68.6)	22 (45.6)		

**Table 6:** Genotyping results on polymorphisms of selected genes (n = 92).

Gene	Polymorphism	Reference Number	Genotype	Total (%)
*VDR*	TaqI	rs731236	A/A	52 (56.5)
			A/G	37 (40.2)
			G/G	3 (3.3)
	BsmI	rs1544410	T/T	53 (57.6)
			T/C	35 (38.0)
			C/C	4 (4.4)
	ApaI	rs7975232	C/C	30 (32.6)
			A/C	52 (56.5)
			A/A	10 (10.9)
	FokI	rs2228570	G/G	44 (47.8)
			G/A	36 (39.2)
			A/A	12 (13.0)

VDR: vitamin D receptor.

**Table 7:** Genetic association of polymorphisms of the *VDR* gene with vitamin D status (*n* = 92).

Polymorphisms	Model	Genotype	Deficiency/Insufficiency vs. Sufficiency				Deficiency vs. Insufficiency/Sufficiency			
			Serum 25(OH)D, ng/mL		OR (95% CI) *	*p*-value	Serum 25(OH)D, ng/mP		OR (95% CI) *	*p*-value
			<30 (*n* = 58)	≥30 (*n* = 34)			<20 (*n* = 35)	≥20 (*n* = 57)		
*VDR* TaqI	CD	A/A	29 (50%)	23 (67.7%)	1.00	0.18	17 (48.6%)	35 (61.4%)	1.00	0.11
		A/G	27 (46.5%)	10 (29.4%)	0.41 (0.15–1.11)		18 (51.4%)	19 (33.3%)	0.49 (0.19–1.27)	
		G/G	2 (3.5%)	1 (2.9%)	0.34 (0.02–6.94)		0 (0%)	3 (5.3%)	NA (0.00–NA)	
	Dom	A/A	29 (50%)	23 (67.7%)	1.00	0.06	17 (48.6%)	35 (61.4%)	1.00	0.23
		A/G-G/G	29 (50%)	11 (32.4%)	0.41 (0.15–1.07)		18 (51.4%)	22 (38.6%)	0.57 (0.22–1.45)	
	Rec	A/A-A/G	56 (96.5%)	33 (97.1%)	1.00	0.63	35 (100%)	54 (94.7%)	1.00	0.13
		G/G	2 (3.5%)	1 (2.9%)	0.50 (0.03–9.32)		0 (0%)	3 (5.3%)	NA (0.00–NA)	
	OD	A/A-G/G	31 (53.5%)	24 (70.6%)	1.00	0.09	17 (48.6%)	38 (66.7%)	1.00	0.1
		A/G	27 (46.5%)	10 (29.4%)	0.43 (0.16–1.15)		18 (51.4%)	19 (33.3%)	0.45 (0.17–1.17)	
*VDR* BsmI	CD	G/G	30 (51.7%)	23 (67.7%)	1.00	0.21	17 (48.6%)	36 (63.2%)	1.00	0.33
		G/A	25 (43.1%)	10 (29.4%)	0.46 (0.17–1.25)		17 (48.6%)	18 (31.6%)	0.49 (0.18–1.28)	
		A/A	3 (5.2%)	1 (2.9%)	0.24 (0.02–3.67)		1 (2.9%)	3 (5.3%)	0.99 (0.09–10.70)	
	Dom	G/G	30 (51.7%)	23 (67.7%)	1.00	0.09	17 (48.6%)	36 (63.2%)	1.00	0.18
		G/A-A/A	28 (48.3%)	11 (32.4%)	0.44 (0.17–1.15)		18 (51.4%)	21 (36.8%)	0.52 (0.20–1.34)	
	Rec	G/G-G/A	55 (94.8%)	33 (97.1%)	1.00	0.38	34 (97.1%)	54 (94.7%)	1.00	0.81
		A/A	3 (5.2%)	1 (2.9%)	0.33 (0.02–4.72)		1 (2.9%)	3 (5.3%)	1.32 (0.13–13.76)	
	OD	G/G-A/A	33 (56.9%)	24 (70.6%)	1.00	0.17	18 (51.4%)	39 (68.4%)	1.00	0.14
		G/A	25 (43.1%)	10 (29.4%)	0.51 (0.19–1.35)		17 (48.6%)	18 (31.6%)	0.49 (0.19–1.26)	
*VDR* ApaI	CD	C/C	18 (31%)	12 (35.3%)	1.00	0.26	11 (31.4%)	19 (33.3%)	1.00	0.96
		A/C	32 (55.2%)	20 (58.8%)	0.74 (0.27–2.04)		21 (60%)	31 (54.4%)	0.90 (0.32–2.51)	
		A/A	8 (13.8%)	2 (5.9%)	0.22 (0.03–1.52)		3 (8.6%)	7 (12.3%)	1.09 (0.21–5.74)	
	Dom	C/C	18 (31%)	12 (35.3%)	1.00	0.36	11 (31.4%)	19 (33.3%)	1.00	0.88
		A/C-A/A	40 (69%)	22 (64.7%)	0.63 (0.24–1.69)		24 (68.6%)	38 (66.7%)	0.93 (0.34–2.51)	
	Rec	C/C-A/C	50 (86.2%)	32 (94.1%)	1.00	0.12	32 (91.4%)	50 (87.7%)	1.00	0.84
		A/A	8 (13.8%)	2 (5.9%)	0.27 (0.05–1.62)		3 (8.6%)	7 (12.3%)	1.17 (0.25–5.41)	
	OD	C/C-A/A	26 (44.8%)	14 (41.2%)	1.00	0.94	14 (40%)	26 (45.6%)	1.00	0.79
		A/C	32 (55.2%)	20 (58.8%)	1.03 (0.41–2.63)		21 (60%)	31 (54.4%)	0.88 (0.34–2.27)	
*VDR* FokI	CD	G/G	30 (51.7%)	14 (41.2%)	1.00	**<0.01**	19 (54.3%)	25 (43.9%)	1.00	0.44
		A/G	25 (43.1%)	11 (32.4%)	**0.92 (0.31–2.67)**		13 (37.1%)	23 (40.4%)	1.07 (0.39–2.96)	
		A/A	3 (5.2%)	9 (26.5%)	**8.90 (1.80–43.97)**		3 (8.6%)	9 (15.8%)	2.59 (0.55–12.21)	
	Dom	G/G	30 (51.7%)	14 (41.2%)	1.00	0.3	19 (54.3%)	25 (43.9%)	1.00	0.55
		A/G-A/A	28 (48.3%)	20 (58.8%)	1.65 (0.64–4.28)		16 (45.7%)	32 (56.1%)	1.33 (0.52–3.43)	
	Rec	G/G-A/G	55 (94.8%)	25 (73.5%)	1.00	**<0.01**	32 (91.4%)	48 (84.2%)	1.00	0.2
		A/A	3 (5.2%)	9 (26.5%)	**9.25 (2.01–42.51)**		3 (8.6%)	9 (15.8%)	2.51 (0.57–11.03)	
	OD	G/G-A/A	33 (56.9%)	23 (67.7%)	1.00	0.25	22 (62.9%)	34 (59.6%)	1.00	0.78
		A/G	25 (43.1%)	11 (32.4%)	0.56 (0.21–1.51)		13 (37.1%)	23 (40.4%)	0.87 (0.33–2.29)	

* Adjusted by age, BMI and sporting experience. CD: codominant; Dom: dominant; NA: not applicable; OD: overdominant; Rec: recessive; VDR: vitamin D receptor.

## Data Availability

The data presented in this study are available on request from the corresponding author. The data are not publicly available due to ethical and privacy restrictions indicated in the informed consent.

## Supplementary Materials

The following supporting information can be downloaded at: https://www.mdpi.com/article/10.3390/nu17203195/s1, Supplementary Material S1: Epidemiological data and vitamin D status of study participants by sports types; Supplementary Material S2: Frequency values according to the Hardy–Weinberg equilibrium test for VDR gene polymorphisms.
