# Supplementary material for: FokI Polymorphism of the VDR Gene Is Associated with Vitamin D Insufficiency in Elite Male Power Athletes of Kazakhstan
Source: Nutrients. 2025 Oct 11;17(20):3195. doi: 10.3390/nu17203195 (PMC12567381; doi:10.3390/nu17203195)
Supplement: Supplementary file 1 [file nutrients-17-03195-s001.zip › Supplementary material S1 Detailed data on vitamin D levels, age, BMI, and years of training for individual sports.pdf]

Epidemiological data and vitamin D status of study participants by sports types

Sport types	*n*	Serum 25(OH)D levels, ng/ml			Age, years		Experience, years	
		< 20	20 ≥ < 30	≥ 30	< 25	≥ 25	< 13	≥ 13
		n (%)	*n* (%)	*n* (%)	*n* (%)	*n* (%)	*n* (%)	*n* (%)
**Boxing**	32	16 (50.0)	11 (34.4)	5 (16.3)	26 (81.3)	6 (18.7)	25 (76.1)	7 (23.9)
**Judo**	23	2 (8.7)	11 (47.8)	10 (44.5)	8 (34.8)	15 (65.2)	8 (34.8)	15 (65.2)
**Freestyle Wrestling**	14	1 (7.1)	7 (50.0)	6 (42.9)	5 (35.7)	9 (64.3)	5 (35.7)	9 (64.3)
**Taekwondo**	10	5 (50.0)	3 (30.0)	2 (20.0)	7 (70.0)	3 (30.0)	7 (70.0)	3 (30.0)
**Greco-Roman Wrestling**	9	7 (77.8)	2 (22.2)	0 (0.00)	5 (55.6)	4 (44.4)	3 (33.3)	6 (66.7)
**Weightlifting**	4	0 (0.00)	1 (25.0)	3 (0.75)	3 (0.75)	1 (25.0)	3 (0.75)	1 (25.0)
