# Supplementary material for: FokI Polymorphism of the VDR Gene Is Associated with Vitamin D Insufficiency in Elite Male Power Athletes of Kazakhstan
Source: Nutrients. 2025 Oct 11;17(20):3195. doi: 10.3390/nu17203195 (PMC12567381; doi:10.3390/nu17203195)
Supplement: Supplementary file 1 [file nutrients-17-03195-s001.zip › Supplementary material S2 HWE.pdf]

**Frequency values according to the Hardy–Weinberg equilibrium test for VDR gene polymorphisms**

| Polymorphism | Groups | Genotypes | | | Alleles | | P value |
|---|---|---|---|---|---|---|---|
| | | A/A | A/G | G/G | A | G | |
| VDR TaqI | All subjects | 52 | 37 | 3 | 141 | 43 | 0.38 |
| | < 30 | 29 | 27 | 2 | 85 | 31 | 0.31 |
| | ≥30 | 23 | 10 | 1 | 56 | 12 | 1 |
| | | G/G | G/A | A/A | G | A | |
| VDR BsmI | All subjects | 53 | 35 | 4 | 141 | 43 | 0.77 |
| | < 30 | 30 | 25 | 3 | 85 | 31 | 0.74 |
| | ≥30 | 23 | 10 | 1 | 56 | 12 | 1 |
| | | A/A | A/G | G/G | A | G | |
| VDR ApaI | All subjects | 30 | 52 | 10 | 112 | 72 | 0.12 |
| | < 30 | 18 | 32 | 8 | 68 | 48 | 0.42 |
| | ≥30 | 12 | 20 | 2 | 44 | 24 | 0.14 |
| | | G/G | G/A | A/A | G | A | |
| VDR FokI | All subjects | 44 | 36 | 12 | 124 | 60 | 0.34 |
| | < 30 | 30 | 25 | 3 | 85 | 31 | 0.74 |
| | ≥30 | 14 | 11 | 9 | 39 | 29 | 0.076 |

VDR: vitamin D receptor

| Polymorphism | Groups | Genotypes | | | Alleles | | P value |
|---|---|---|---|---|---|---|---|
| | | A/A | A/G | G/G | A | G | |
| VDR TaqI | All subjects | 52 | 37 | 3 | 141 | 43 | 0.38 |
| | < 20 | 17 | 18 | 0 | 52 | 18 | 0.074 |
| | ≥20 | 35 | 19 | 3 | 89 | 25 | 0.72 |
| | | G/G | G/A | A/A | G | A | |
| VDR BsmI | All subjects | 53 | 35 | 4 | 141 | 43 | 0.77 |
| | < 20 | 17 | 17 | 1 | 51 | 19 | 0.39 |
| | ≥20 | 36 | 18 | 3 | 90 | 24 | 0.69 |
| | | A/A | A/G | G/G | A | G | |
| VDR ApaI | All subjects | 30 | 52 | 10 | 112 | 72 | 0.12 |
| | < 20 | 11 | 21 | 3 | 43 | 27 | 0.17 |
| | ≥20 | 19 | 31 | 7 | 69 | 45 | 0.41 |
| | | G/G | G/A | A/A | G | A | |
| VDR FokI | All subjects | 44 | 36 | 12 | 124 | 60 | 0.34 |
| | < 20 | 19 | 13 | 3 | 51 | 19 | 0.68 |
| | ≥20 | 25 | 23 | 9 | 73 | 41 | 0.39 |

VDR: vitamin D receptor
